# Pulmonary Dysfunction in COPD

**DOI:** 10.1155/2013/535820

**Published:** 2013-04-08

**Authors:** Kostas Spiropoulos, Kiriakos Karkoulias, Nikolaos Koulouris, Edgardo D'Angelo

**Affiliations:** ^1^Department of Pulmonary Medicine, University Hospital of Patras, University of Patras, Rio, 26500 Patras, Greece; ^2^Department of Respiratory Medicine, “Sotiria” Hospital for Diseases of the Chest, Nathional and Kapodistrian University of Athens, 11527 Athens, Greece; ^3^Dipartimento di Fisiopatologia Medico-chirurgica e dei Trapianti, Università degli Studi di Milano, 20133 Milan, Italy

Chronic obstructive pulmonary disease (COPD) is a major cause of morbidity and mortality all around the world. It has been identified as the fourth leading cause, which will rise globally to the third place, before the end of 2020. It is estimated that more than 12 million adults suffer from COPD in the USA and 24 million patients have an impaired lung function, which may lead to the development of COPD. Tobacco smoke is the predominant but not the only environmental risk factor for COPD. COPD is a burden for health providing systems as the estimated direct and indirect costs are constantly raising.

Chronic obstructive pulmonary disease, namely, pulmonary emphysema and chronic bronchitis, is a chronic inflammatory response of the airways to noxious particles or gases, with resulting pathological and pathophysiological changes in the lung. The main pathophysiological aspects of the disease are airflow obstruction and hyperinflation, which are discussed by D. Papandrinopoulou et al. The mechanical properties of the respiratory system and its component parts are studied by determining the corresponding volume-pressure (V-P) relationships. The consequences of the inflammatory response on the lung structure and function are depicted on the volume-pressure relationships.

Expiratory flow limitation is well discussed by Tantucci. When expiratory flow is maximal during tidal breathing and cannot be increased unless operative lung volumes move towards total lung capacity, tidal expiratory flow limitation (EFL) is said to occur. In any circumstances, EFL predisposes to pulmonary dynamic hyperinflation and its unfavorable effects such as increased elastic work of breathing, inspiratory muscles dysfunction, and progressive neuroventilatory dissociation, leading to reduced exercise tolerance, marked breathlessness during effort, and severe chronic dyspnea.

N. G. Koulouris et al. in their paper discuss the expiratory flow limitation in COPD patients at rest (EFLT). EFLT, namely, attainment of maximal expiratory flow during tidal expiration, occurs when an increase in transpulmonary pressure causes no increase in expiratory flow. EFLT leads to small airway injury and promotes dynamic pulmonary hyperinflation with concurrent dyspnea and exercise limitation. Among the currently available techniques, the negative expiratory pressure (NEP) has been validated in a wide variety of settings and disorders. Consequently, it should be regarded as a simple, noninvasive, most practical, and accurate new technique.

COPD is a complex pathological condition associated with an important reduction in physical activity and psychological problems that contribute to the patient's disability and poor health-related quality of life as it is stated in the paper of P. Santus et al. Pulmonary rehabilitation is aimed to eliminate or at least attenuate these difficulties, mainly by promoting muscular reconditioning. Pulmonary rehabilitation has a beneficial effect on dyspnea relief, improving muscle strength and endurance. Moreover, it appears to be a highly effective and safe treatment for reducing hospital admissions, mortality, and improving health-related quality of life in COPD patients.

The paper of F. Krakontaki et al. attempts to show the impact of COPD on the cognitive functions of the patients. The findings provide evidence that stable COPD patients may manifest impaired information processing operations. Therefore, COPD patients should be warned of the potential danger and risk they face when they drive any kind of vehicle, even when they do not exhibit overt symptoms related to driving ability.

The deterioration of quality of life of COPD smokers is illustrated by S. Joseph et al. in a study population from Lebanon. The Clinical COPD Questionnaire (CCQ) demonstrated excellent psychometric properties, with a very good adequacy to a cross-sectional sample and high consistency. Smokers had a decreased respiratory quality of life versus nonsmokers, independently of their respiratory disease status and severity.

I. Tsangaris et al. show one of the most important complications of chronic hypoxemia in COPD, which is pulmonary hypertension. Interestingly, in types of PH that are encountered in parenchymal lung diseases such as interstitial lung diseases (ILDs), chronic obstructive pulmonary disease (COPD), and many other diffuse parenchymal lung diseases, some of which are very common, the available data is limited. The paper summarizes the latest available data regarding the occurrence, pathogenesis, and treatment of PH in chronic parenchymal lung diseases.

Finally, M. Pecchiari discusses the role of heliox, which has been administered to stable chronic obstructive pulmonary disease (COPD) patients at rest and during exercise on the assumption that this low density mixture would have reduced work of breathing, dynamic hyperinflation, and, consequently, dyspnea sensation. Contrary to these expectations, beneficial effects of heliox in these patients at rest have been reported only sporadically. On the other hand, when it is administered to COPD patients exercising at a constant work rate, heliox systematically decreases dyspnea sensation and, often but not always, increases exercise tolerance. Therefore, further studies, aimed to the identification of mechanisms conditioning the response of exercising COPD patients to heliox, are warranted, before heliox administration, which is costly and cumbersome, can be routinely used in rehabilitation programs.

